# Use of sulfasalazine for psoriasis: An evidence-based review

**DOI:** 10.1016/j.jdin.2024.02.012

**Published:** 2024-02-24

**Authors:** Siddhartha Sood, Ahmed Bagit, Khalad Maliyar, Muskaan Sachdeva, David Croitoru, Jensen Yeung

**Affiliations:** a Temerty Faculty of Medicine, University of Toronto, Toronto, Ontario, Canada; b Division of Dermatology, Department of Medicine, University of Toronto, Toronto, Ontario, Canada; c Department of Dermatology, Sunnybrook Health Sciences Centre, Toronto, Ontario, Canada; d Department of Dermatology, Women's College Hospital, Toronto, Ontario, Canada; e Probity Medical Research, Waterloo, Ontario, Canada

**Keywords:** evidence-based, immunomodulator, psoriasis, sulfasalazine, systematic review

*To the Editor:* Psoriasis is a chronic T-helper (Th)-1/Th-17 mediated inflammatory disease which often requires systemic therapy for skin clearance.^1^^,^^2^ Sulfasalazine is an antiinflammatory medication comprised of sulphapyridine and 5-aminosalicylic acid and is used in many immune-mediated diseases.^3^^,^^4^ Despite having a favorable efficacy and safety profile demonstrated in small randomized trials, it is currently off-label for treatment of psoriatic arthritis and plaque psoriasis in North America.^1^^,^^3^ We performed a systematic review to assess outcomes of sulfasalazine for cutaneous psoriasis.

We followed Preferred Reporting Items for Systematic Reviews and Meta-Analyses guidelines to search Embase and MEDLINE databases using several keywords (Supplementary Table I, available via Mendeley at https://doi.org/10.17632/34d9fjjng8.1). Quality of evidence was assessed using Oxford Centre for Evidence-Based Medicine 2011 Levels of Evidence. Original articles written in English reflecting an observational or experimental design were included. After independent screening by 2 reviewers, 10 articles encompassing 246 patients were included (Fig 1; Supplementary Table II, available via Mendeley at https://doi.org/10.17632/34d9fjjng8.1). The mean age was 24.1 years (range: 16-74 years) with sex reported in 176 (71.5%) instances (male: 72.7%, 128/176; female: 27.3%, 48/176). Comorbid psoriatic arthritis was present in 187/246 (76%) cases. Refractory disease to non-sulfasalazine systemic therapy was noted in 45.9% (113/246) of patients.

**Fig 1:**
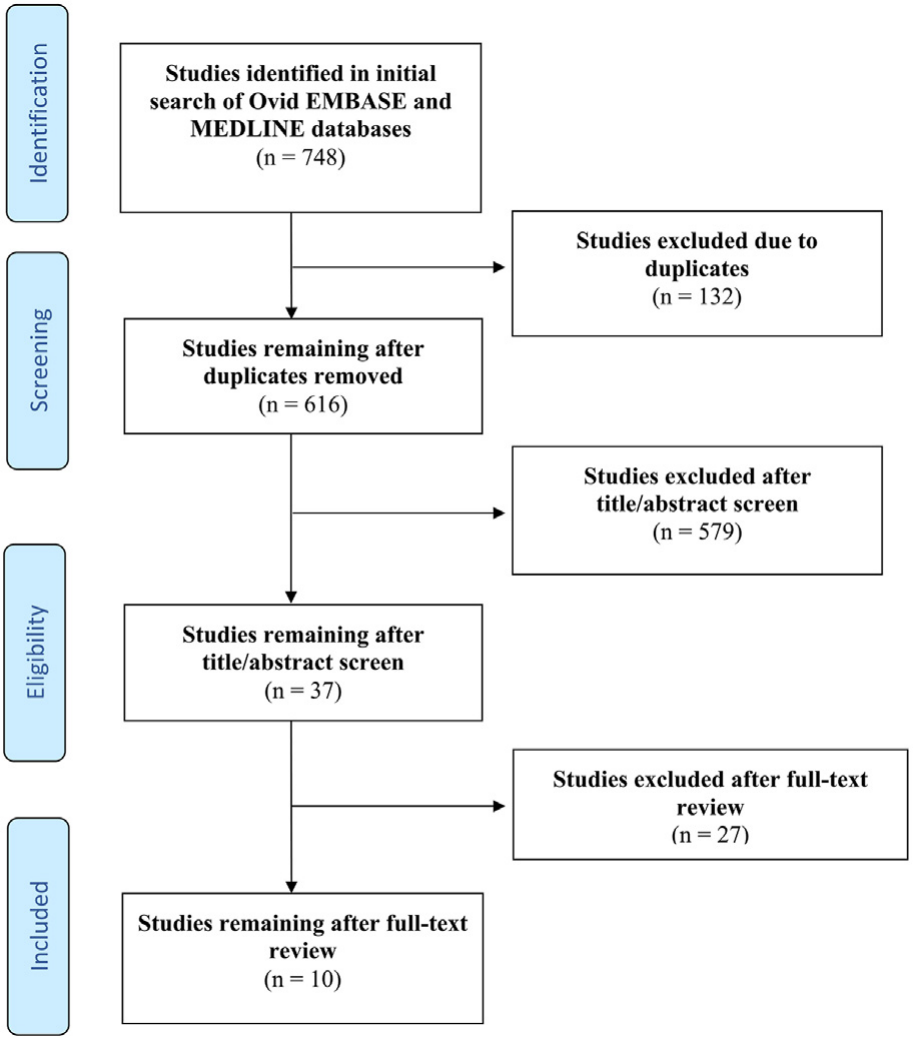
Flow diagram of literature screening using the preferred reporting items for systematic reviews and meta-analyses guidelines. Figure adapted from http://prisma-statement.org.

The mean treatment duration was 18.4 weeks (range: 1.6-36 weeks). The most common daily dose of sulfasalazine was 2 g (82.5%, 203/246) (range: 1.5 g-4 g). Concomitant medications were used in 12.6% (31/246) of patients with frequent use of nonsteroidal antiinflammatory drugs (51.6%, 16/31) (Supplementary Table II, available via Mendeley at https://doi.org/10.17632/34d9fjjng8.1). In 8.9% (22/246) of patients, there was a reported complete clearance, and partial resolution was seen in 44.7% (110/246) of cases. No improvement was documented in 46.3% (114/246) of instances (Table I). In 49/246 cases where Psoriasis Area and Severity Index (PASI) was reported, sulfasalazine use led to a mean improvement of 48.4% (range: 47%-54.7%) within 18.4 weeks, including 5/49 (10.2%) and 2/49 (4.1%) patients achieving PASI-50 and PASI-75 respectively. In 158/246 cases where body surface area was reported, a reduction of 18.5% (range: 9.3%-66.7%) was seen. There were 87/264 (35.4%) treatment-emergent adverse events reported, commonly reflecting gastrointestinal intolerance (26.4%, 23/246) and nausea (17.2%, 15/246). In total, there were 40/246 (16.3%) discontinuations with 5/264 (1.9%) severe adverse events (Supplementary Table II, available via Mendeley at https://doi.org/10.17632/34d9fjjng8.1).

**Table I:** Summary of sulfasalazine outcomes for psoriasis

Study design (*n*/*N*, %)	
Randomized controlled trial	5/10 (50%)
Case report	3/10 (30%)
Prospective cohort study	2/10 (20%)
PASI	
PASI: mean (reported cases)	−48.4% (49/246)
PASI: range	−47% to −54.7%
PASI-50 responders (*n*/*N*, %)	5/49 (10.2%)
PASI-75 responders (*n*/*N*, %)	2/49 (4.1%)
BSA	
BSA: mean (reported cases)	−18.5% (158/246)
BSA: range	−9.3% to −66.7%
Treatment outcome	
CC (*n*/*N*, %)	22/246 (8.9%)
Mean treatment duration for CC (reported cases) (wk)	16.3 (22/22)
Mean follow-up period for CC (reported cases) (mo)	6.1 (22/22)
PR (*n*/*N*, %)	110/246 (44.7%)
Mean treatment duration for PR (reported cases) (wk)	26.4 (110/110)
Mean follow-up period for PR (reported cases) (mo)	30.2 (110/110)
NIM (*n*/*N*, %)	114/246 (46.3%)
Mean treatment duration for NIM (reported cases) (wk)	29 (114/114)
Mean follow-up period for NIM (reported cases) (mo)	30.5 (114/114)
Treatment characteristics	
Treatment duration (wk): mean	18.4
Treatment duration (wk): range	1.6 to 36
Follow-up period (mo): mean	27.4
Follow-up period (mo): range	0.25 to 168
Recurrence rate (*n*/*N*, %)	8/132 (6.1%)
Adverse events (*n*/*N*, %)	
Gastrointestinal intolerance	23/246 (9.3%)
Nausea	15/246 (6.1%)
Unspecified cutaneous eruption	9/246 (3.7%)
Neurological	7/246 (2.8%)
Fatigue	6/246 (2.4%)
Increased liver enzymes	6/246 (2.4%)
Heartburn	3/246 (1.2%)
Loss of taste	2/246 (0.8%)
Photosensitive eruption	2/246 (0.8%)
Purpura	2/246 (0.8%)
Hypertension	1/246 (0.4%)
Erythrodermic eruption∗	1/246 (0.4%)
Fever	1/246 (0.4%)
Impaired renal function∗	1/246 (0.4%)
Leukopenia	1/246 (0.4%)
Lymphadenopathy	1/246 (0.4%)
Macular eruption	1/246 (0.4%)
Nocturia	1/246 (0.4%)
Systemic fever, rash, and leukopenia∗	1/246 (0.4%)
Thrombocytopenia∗	1/246 (0.4%)
Toxic epidermal necrolysis∗	1/246 (0.4%)

*BSA*, Body surface area; *CC*, complete clearance; *NIM*, no improvement; *PASI*, Psoriasis Area and Severity Index; *PR*, partial resolution.

∗ Severe adverse events.

While the mechanism of action of sulfasalazine remains unclear, studies have demonstrated its inhibition of nuclear factor-κB activation in dendritic cells as well as Th1-related cytokines.^4^^,^^5^ Notably, we found 53.6% (132/246) of patients in this review improved or cleared on sulfasalazine, with few serious adverse events. Currently, sulfasalazine is outlined as an option for first-line conventional disease-modifying antirheumatic drug therapy in peripheral psoriatic arthritis.^3^ Recent joint American Academy of Dermatology-National Psoriasis Foundation psoriasis guidelines do not provide a recommendation on sulfasalazine use.

Study limitations include a small sample size of reported PASI data, incomplete follow-up data, and publication bias. While available efficacy findings are modest in comparison to other available psoriasis drugs, this review demonstrates sulfasalazine may provide an accessible low-cost option for a subset of psoriasis patients that have contraindications to other systemic agents or are refractory to first-line systemic immunosuppressives and biologics.^1^ Prospective studies should be conducted in special populations.

## Conflicts of interest

Dr Yeung has been an advisor, consultant, speaker, and/or investigator for AbbVie, Amgen, Anacor, Arcutis, Astellas, Bausche, Baxalta, Boehringer Ingelheim, Bristol Myers Squibb, Celgene, Centocor, Coherus, Dermira, Forward, Fresenius Kabi, Galderma, Incyte, Janssen, LEO Pharma, Medimmune, Merck, Novartis, Pfizer, Regeneron, Roche, Sanofi Genzyme, Sun Pharma, Takeda, UCB, and Xenon. Authors Sood, Bagit, Drs Maliyar, Sachdeva, and Croitoru have no conflicts of interest to declare.
